# Effects of Naked Gold Nanoparticles on Proinflammatory Cytokines mRNA Expression in Rat Liver and Kidney

**DOI:** 10.1155/2013/590730

**Published:** 2013-05-26

**Authors:** Haseeb A. Khan, Mohamed Anwar K. Abdelhalim, Abdullah S. Alhomida, Mohammed S. Al-Ayed

**Affiliations:** ^1^Department of Biochemistry, College of Science, King Saud University, P.O. Box 2455, Riyadh 11451, Saudi Arabia; ^2^Department of Physics and Astronomy, College of Science, King Saud University, P.O. Box 2455, Riyadh 11451, Saudi Arabia

## Abstract

The data on the biocompatibility of naked gold nanoparticles (GNPs) are scarce, and their interpretation is controversial. We studied the acute (1 day) and subchronic (5 days) effects of GNPs (10 and 50 nm diameter) on expression of interleukin-1 beta (IL-1**β**), interleukin-6 (IL-6), and tumor necrosis factor-alpha (TNF-**α**) in the liver and kidneys of rats. In the liver, the GNPs of both sizes (10 and 50 nm) significantly increased the cytokines gene expression on day 1 which was subsided on day 5; the GNPs of 50 nm size produced more severe inflammatory response as compared to smaller sized GNPs. In the kidney, the GNPs did not produce any significant change in the expression of IL-1**β**. Although the gene expression of IL-6 and TNF-**α** was not affected by GNPs of 10 nm size, 50 nm GNPs significantly increased the expression of IL-6 and TNF-**α** in the kidneys of rats on day 1 after treatment which returned to normalcy on day 5. These findings indicate the possible immunocompatibility of medium sized GNPs as they caused only a transient acute phase increase in proinflammatory cytokines expression followed by their normalcy during the repeated exposure.

## 1. Introduction

Gold nanoparticles (GNPs) possess the promising therapeutic possibilities due to their unique properties such as biocompatibility, high surface reactivity, resistance to oxidation, flexibility in functionalization, and a wide range of delivery targets [1–3]. GNPs have been found to be useful for the delivery and controlled release of a variety of chemical agents including anticancer drugs [4], antibiotics [5], amino acids [6], peptides [7], glucose [8], antioxidants [9], nucleic acids [10], and isotopes [11]. The promise of GNPs for different biological applications has led to a strong interest in studying their possible deleterious effects in biological systems and how these effects might be mitigated.

Most of the previous studies on GNPs were conducted on polyethylene glycol- (PEG-) coated GNPs rather than the naked or unmasked GNPs. PEG is known to improve the circulation half life of particles by creating a steric shield thereby effectively preventing plasma proteins from adhering to their surface [12, 13]. *In vivo* biodistribution study in a mice model bearing subcutaneously inoculated U14 tumor has confirmed that 12.1 and 27.3 nm PEG-coated GNPs are accumulated in the tumor with high concentrations and do not cause spleen and kidney damages but give rise to liver damage and gold accumulation [14]. PEG-coated 13 nm sized GNPs have been found to accumulate in the liver and spleen of mice for up to 7 days after a single intravenous (IV) injection and induced acute inflammation and apoptosis in the liver [15]. Intravenously administered PEG-Silicon GNPs elicited a mild inflammatory response and increased oxidative stress in the liver after 24 h, which subsided by 2 weeks after dosing without causing any significant toxicity [16]. Morais et al. [17] evaluated the biodistribution of 20 nm GNPs with six different surface coatings and demonstrated that GNPs are rapidly distributed while liver is the preferential accumulation organ with GNPs trapped in Kupffer cells, hepatocytes, and endosomes. The hepatic uptake of GNPs was significantly increased by peptide capping [17].

The major determinant factors to modify biodistribution, toxicity, and biocompatibility of GNPs include their size, shape, charge, and surface modifications. Recently, 50 nm GNPs have been found to be more effective against MCF7 breast cells in terms of superior penetration in cultured cells and accumulated more effectively in tumor xenografts *in vivo* after a single intravenous dose [18]. By contrast, larger GNPs were primarily localized in the periphery of the tumor spheroid and around blood vessels, hindering their deep penetration into tumors [18]. A biodistribution study showed that 5 nm and 10 nm PEG-coated GNPs accumulated in the liver and 30 nm particles accumulated in the spleen, while the 60 nm particles did not accumulate to an appreciable extent in either organ of mice suggesting that the toxicity of PEG-coated GNPs is complex and it cannot be concluded that the smaller particles have greater toxicity and vice versa [19]. Gold nanospheres (20 nm diameter) coated with mercaptopropane sulfonate were found to be nontoxic in human keratinocyte cell line whereas gold nanorods (16.7 nm diameter and 43.8 nm long) coated with PEG caused significant generation of reactive oxygen species (ROS) and upregulation of genes involved in cellular stress and toxicity suggesting that shape appears to play a key role in mediating the cellular response to GNPs [20]. The systemic toxicity of the intermediate sized (18–37 nm) citrate-capped GNPs was linked to major organ damage in the liver, spleen, and lungs of mice; however the same nanoparticles were found to be nontoxic *in vitro* using HeLa cell lines [21]. It is crucially important to investigate the *in vivo* effects of nanomaterials before approving any potential therapeutic applications [22]. In this investigation, we studied the time-course effects of 10 and 50 nm sized naked GNPs on the expression of proinflammatory cytokines including IL-1*β*, IL-6, and TNF-*α* in the liver and kidneys of rats.

## 2. Materials and Methods

### 2.1. Animals and Treatment Groups

Adult male Wistar-Kyoto rats, weighing 210–250 g, were obtained from the Laboratory Animal Centre, College of Pharmacy, King Saud University, Riyadh. The animals were housed in humidity- and temperature-controlled ventilated cages on a 12 h day/night cycle, with free access to standard laboratory food and tap water. The animals were randomly divided into 5 groups of 5 animals each. One group served as control and received vehicle only. Two groups were treated with GNPs (10 nm diameter) for 1 and 5 days, respectively. The remaining two groups received GNPs (50 nm diameter) for 1 and 5 days, respectively.

### 2.2. Gold Nanoparticles (GNPs)

Gold nanoparticles of 10 nm diameter (MKN-Au-010 of concentration 0.01% Au) and 50 nm (MKN-Au-050 of concentration 0.01% Au) were purchased from MK Impex Corp., ON, Canada. The morphology of these GNPs was evaluated by transmission electron microscopy (TEM) images showing that 10 nm GNPs are round shaped, but 50 nm GNPs are hexagonal (Table 1). 

### 2.3. Animal Dosing

Doses of 50 *μ*L of 10 nm and 50 nm GNPs in aqueous solution were administered to animals via intraperitoneal (IP) injection daily for 1 or 5 days. This dosage is approximately equivalent to 22 *μ*g Au/kg bodyweight of rat. The dose regimen is summarized in Table 1. The rats were sacrificed 24 h after the last injection of GNPs. The specimens of liver and kidney were isolated and immediately immersed in RNAlater solution (Qiagen) and stored at 4°C until RNA extraction. All experiments were conducted in accordance with guidelines approved by our Institutional Animal Care and Use Committee.

### 2.4. qRT-PCR

Expressions of mRNAs for the proinflammatory cytokines, IL-1*β*, IL-6, and TNF-*α* were quantified by real-time qRT-PCR. Total RNA was isolated from liver and kidney tissues (approximately 30 mg) using RNAEasy kit (Qiagen, Germany), according to the manufacturer's protocol. The extracted RNA was dissolved in 30 *μ*L of nuclease free distilled water and stored at −20°C. The concentration and purity of RNA were determined by Nanodrop Spectrophotometer (Thermo Scientific). Real-time PCR was performed using 2 *μ*L of template in a 20 *μ*L reaction containing 0.25 *μ*M of each primer and 12.5 *μ*L Sybr Green real-time PCR MasterMix (Applied Biosystems). Each run consisted of 50°C for 2 min and 95°C for 10 min followed by 45 cycles of 95°C for 15 s, 60°C for 20 s, and 72°C for 60 s in a real-time qPCR machine (Stratagene, Agilent Biosciences, or Bio-Rad). A housekeeping gene, glyceraldehyde-3-phosphate dehydrogenase (GAPDH), was used as an external standard for normalizing the expression data [23]. The primers sequences were as follows: IL-1*β* (caccttcttttccttcatctttg; gtcgttgcttgtctctccttgta), IL-6 (tgatggatccttccaaactg; gagcattggaagttggggta), TNF-*α* (actgaacttcggggtgattg; gcttggtggtttgctacgac), and GAPDH (gtattgggcgcctggtcacc; cgctcctggaagatggtgatgg).

### 2.5. Statistics

The data were analyzed by one-way analysis of variance (ANOVA) followed by Dunnett's multiple comparison test using SPSS statistical package. *P* values less than 0.05 were considered as statistically significant.

## 3. Results 

**Figure 1:**
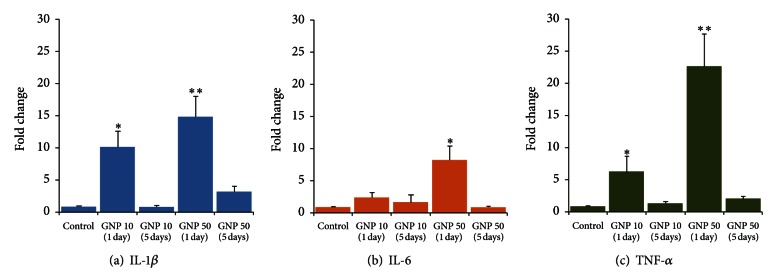
Time-course effects of gold nanoparticles (GNPs) of 10 and 50 nm diameter on proinflammatory cytokines gene expression in rat liver. *P < 0.05 and **P < 0.01 versus control group using Dunnett's test.

There were significant and dose-dependent increases in IL-1*β* mRNA expression in liver on day 1 after dosing of 10 nm (10.14-fold) and 50 nm GNPs (14.81-fold) which were significantly reduced on day 5 (ANOVA *F* = 5.49, *P* < 0.01) (Figure 1). The expression of IL-6 in liver was also dose dependently increased by 10 nm (2.46-fold) and 50 nm (8.26-fold) GNPs after 1 day; however this increase was significant while using 50 nm GNPs (ANOVA *F* = 3.18, *P* < 0.05). IL-6 gene expression was normalized on day 5. TNF-*α* mRNA expression in liver was significantly increased on day 1, by 10 nm (6.34-fold) and 50 nm (22.65-fold) GNPs, which was significantly reduced on day 5 (ANOVA *F* = 3.09, *P* < 0.05) (Figure 1).

**Figure 2:**
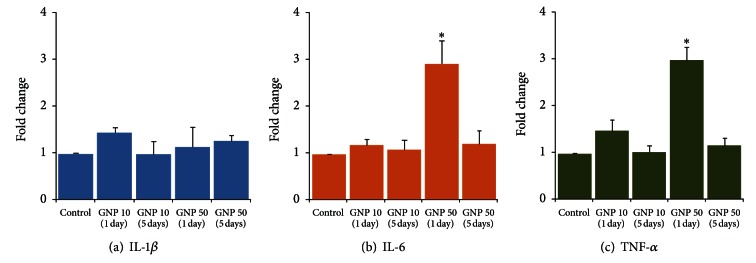
Time-course effects of gold nanoparticles (GNPs) of 10 and 50 nm diameter on proinflammatory cytokines gene expression in rat kidney. *P < 0.01 versus control group using Dunnett's test.

In kidneys, the small sized GNPs (10 nm) did not produce any significant change in the expression of IL-1*β*, IL-6, or TNF-*α* (Figure 2). Although the GNPs of 50 nm diameter failed to alter the expression of IL-1*β* (ANOVA *F* = 0.84, *P* = 0.515), they significantly increased the expression of IL-6 (2.95-fold) (ANOVA *F* = 8.83, *P* < 0.001) and TNF-*α* (3.02-fold) (ANOVA *F* = 22.73, *P* < 0.001) on day 1, which returned to normal levels on day 5 (Figure 2). 

## 4. Discussion

There was a significant increase in proinflammatory cytokines mRNA expression in liver (Figure 1) as compared to kidneys (Figure 2) that can be attributed to variations in the biodistribution and accumulation of GNPs in different organs. Sadauskas et al. [24] have shown intense uptake of GNPs (2 and 40 nm) by the Kupffer cells in the liver following a single IV injection in mice, whereas the IP route resulted in the uptake of GNPs in the Kupffer cells as well as in the macrophages of mesenterial lymph nodes, lymphatic tissue in the wall of the small intestine, and in the spleen [24]. However, there was no accumulation of GNPs in kidneys, brain, lungs, adrenals, and ovaries of both IP and IV injected animals, at 24 h after injection [24]. Altered accumulation of GNPs in various organs and tissues has been related to the GNPs sizes and surface charges that mediated the dynamic protein binding and exchange [25]. Adsorption of plasma proteins onto the surface of nanoparticles, known as opsonization, occurs instantly when the particles enter the blood stream [26]. Opsonization of nanoparticles is a crucial step by which they are recognized and cleared by phagocytosis. Besides opsonins, other proteins in blood such as albumin and apolipoproteins may bind to GNPs and alter their cellular uptake [27]. Sadauskas et al. [24] did not find the accumulation of GNPs in cells other than macrophages suggesting that inert GNPs do not penetrate cell membranes by nonendocytotic mechanisms. However, the presence of nanoparticles in erythrocytes indicates that nanoparticles are also able to cross the cell membrane of erythrocytes by processes other than phagocytosis since erythrocytes do not have phagocytotic receptors [28, 29]. Diffusion, transmembrane channels, adhesive interactions, or other undefined, transmembrane processes might play a role in this cellular uptake [30].

 In rats, the accumulation of GNPs in liver at 24 h after intravenous injection was found to be in the range from 91.9 to 96.9% for 5, 18, 80 and 200 nm GNPs whereas the hepatobiliary clearance of GNPs (from liver to small intestine to fecal excretion) showed an inverse linear relationship to the GNP diameter over the size range of 5 nm to 200 nm [25]. Another study in rats showed that 24 h, after intravenous administration of 20 nm GNPs, the liver exhibited the highest uptake of Au (49.4 ng/g) followed by the spleen (9.5 ng/g), that persisted throughout the entire duration of study (2 months) [31]. On the other hand, kidney showed only slight increase in the amounts of Au (1.0 ng/g) on day 1 but significant increases after 1 month (3.4 ng/g) and 2 months (5.5 ng/g), suggesting that GNPs could gradually be coated with serum proteins that alters their shape, charge, and hydrodynamic diameter hindering their renal clearance [31]. A biodistribution study in mice intravenously exposed to 1 g/kg dose of GNPs revealed that 15 nm GNPs are mainly accumulated in liver (52.26 *μ*g/g) followed by lung (32.27 *μ*g/g), kidney (25.48 *μ*g/g), brain (9.95 *μ*g/g) and spleen (5.46 *μ*g/g) whereas 50 nm GNPs are mainly accumulated in liver (21.25 *μ*g/g) followed by lung (18.65 *μ*g/g), spleen (11.53 *μ*g/g), brain (9.12 *μ*g/g), and kidney (3.75 *μ*g/g) [32]. The above studies clearly indicate that accumulation of GNPs in liver is several folds higher than that in kidneys reflecting the increased expression of proinflammatory cytokines in the former organ. 

 In our study, 50 nm GNPs caused more severe acute phase expression of proinflammatory cytokines as compared to 10 nm GNPs in liver (Figure 1). It is more likely that larger size and hexagonal shape of 50 nm GNPs facilitated the adsorption of plasma proteins resulting in comparatively increased immunoactivation. In kidneys, 10 nm GNPs did not affect the expression of cytokines while 50 nm GNPs significantly increased the expression of IL-6 and TNF-*α* (Figure 2). By virtue of accessibility, smaller NPs tend to have a wider distribution in the body as compared to larger sized NPs. After a single intravenous injection in rats, the 10 nm GNPs were present in various organ systems including blood, liver, spleen, kidney, testis, thymus, heart, lung, and brain, whereas the larger particles were mainly detected in blood, liver, spleen, and kidneys [30]. Moreover, smaller size also favors the renal clearance of GNPs. Urinary excretion of 20 nm GNPs despite comparatively smaller pore size (8 nm) of glomerular basement membrane has been linked to neutral or small charges on GNPs to counteract the repulsive forces and ultimately manage to pass through the filtration barrier [31]. Sun et al. [33] have shown that the *in vivo* toxicity and biodistribution of GNPs in mice are shape dependent; sphere-shaped GNPs displayed the best biocompatibility compared with cube-shaped GNPs, whereas the rod-shaped GNPs were most toxic. PEG-coated gold nanorods were taken up to a lesser extent by the liver and had longer circulation time in the blood and higher accumulation in the tumors, compared with their spherical counterparts pointing the importance of GNPs geometry and surface properties on their transport across biological barriers [12]. 

 In both organs, the proinflammatory cascade returned to normalcy after the repeated exposure of GNPs (Figures 1 and 2). Based upon *in vivo* studies, no toxic effects of 12.5 nm GNP were found in the liver, lungs, kidneys, spleen, or brain [34, 35]. Glazer et al. [36] assessed the acute toxicity and biodistribution of 5 nm and 25 nm naked GNPs in rabbits with implanted liver tumors 24 hours after intravenous injection and did not find any evidence of renal, hepatic, pulmonary, or other organ dysfunction. The findings of a microscopic study suggest that GNPs are not cytotoxic as they reduce the production of ROS and do not elicit secretion of proinflammatory cytokines (TNF-*α* and IL1-*β*) making them suitable candidates for nanomedicine [37]. Zhang et al. [38] have reported that 60 nm GNPs are neither cytotoxic nor elicit proinflammatory responses (IL-6, TNF-*α*) in murine macrophages despite the cellular uptake of GNPs and localization within intracellular vacuoles was evident. Incubation of dendritic cells in the presence of GNPs did not affect the cellular viability and phenotypic morphology despite significant accumulation of GNPs in endocytic compartments; however the secretion of cytokines was significantly modified indicating a potential perturbation of the immune response [39]. Yen et al. [40] have found that both silver and gold NPs enter the cells, but only GNPs upregulate the expressions of proinflammatory genes (IL-1, IL-6, and TNF-*α*). They speculated that part of the negatively charged GNPs might adsorb serum protein and enter cells via the more complicated endocytotic pathway, resulting in higher cytotoxicity and immunological response [40]. Citrate-stabilized and dihydrolipoic acid functionalized GNPs neither induced apoptosis nor activated gene expression related to oxidative stress and inflammatory response (TNF-*α*) while their decreased reactivity with biomolecules and cells provides a promising medical platform [41]. A size-dependent toxic impact of GNP was observed *in vitro* which occurred for 1.4 nm GNP, but not for 15 nm GNP, or 0.8 nm GNP [42]. The findings of the excessive cytotoxicity of the 1.4 nm GNP were explained by the perfect fitting of this GNP in the major grooves of the DNA causing its immobility [35].

In conclusion, naked GNPs-induced expression of proinflammatory cytokines was several folds higher in liver than in kidneys of rats. In liver, 10 and 50 nm GNPs significantly increased cytokines gene expression on day 1 which subsided on day 5. However, the GNPs of 50 nm size produced more severe induction of proinflammatory cytokines as compared to smaller GNPs. In kidneys, GNPs did not produce any significant change in the expression of IL-1*β*. Although the gene expression of IL-6 and TNF-*α* was not affected by 10 nm GNPs, 50 nm GNPs significantly increased the expression of IL-6 and TNF-*α* in the kidneys of rats on day 1, which was normalized on day 5. These findings point towards a possible immunocompatibility of medium sized naked GNPs (10–50 nm) as they caused only a transient increase in proinflammatory cytokines that returned to normal level after the regenerative phase was over. However, the impact of this acute phase inflammatory response on triggering the generation of ROS is a matter of investigation. Further studies are warranted on *in vivo* effects of pure and functionalized GNPs on inflammatory cascade and cellular integrity in different organs systems including lung and spleen.

## Figures and Tables

**Table 1:** Commercial properties of GNPs and the rat doses used in this study.

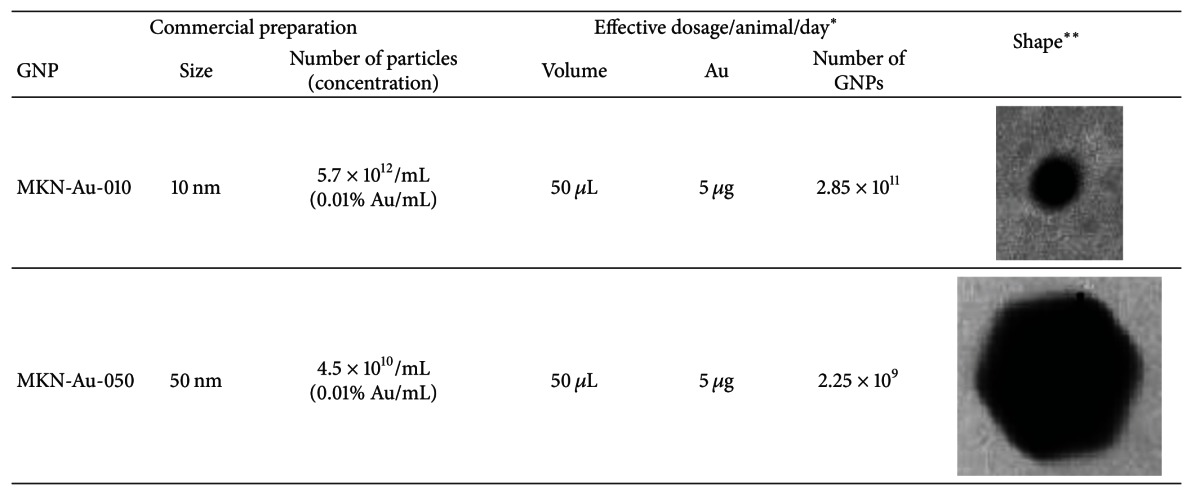

*This dose is approximately equivalent to 22 *μ*g/kg bodyweight of rat. **TEM images showing that the shape of 10 nm GNP is spherical and that of 50 nm GNP is hexagonal.
